# Supplementary material for: Organismal and Cellular Stress Responses upon Disruption of Mitochondrial Lonp1 Protease
Source: Cells. 2022 Apr 16;11(8):1363. doi: 10.3390/cells11081363 (PMC9025075; doi:10.3390/cells11081363)
Supplement: Supplementary file 1 [file cells-11-01363-s001.zip › cells-1658543-supplementary/Revised Supplementary Figures S1-S9.pdf]

Supplementary Figures

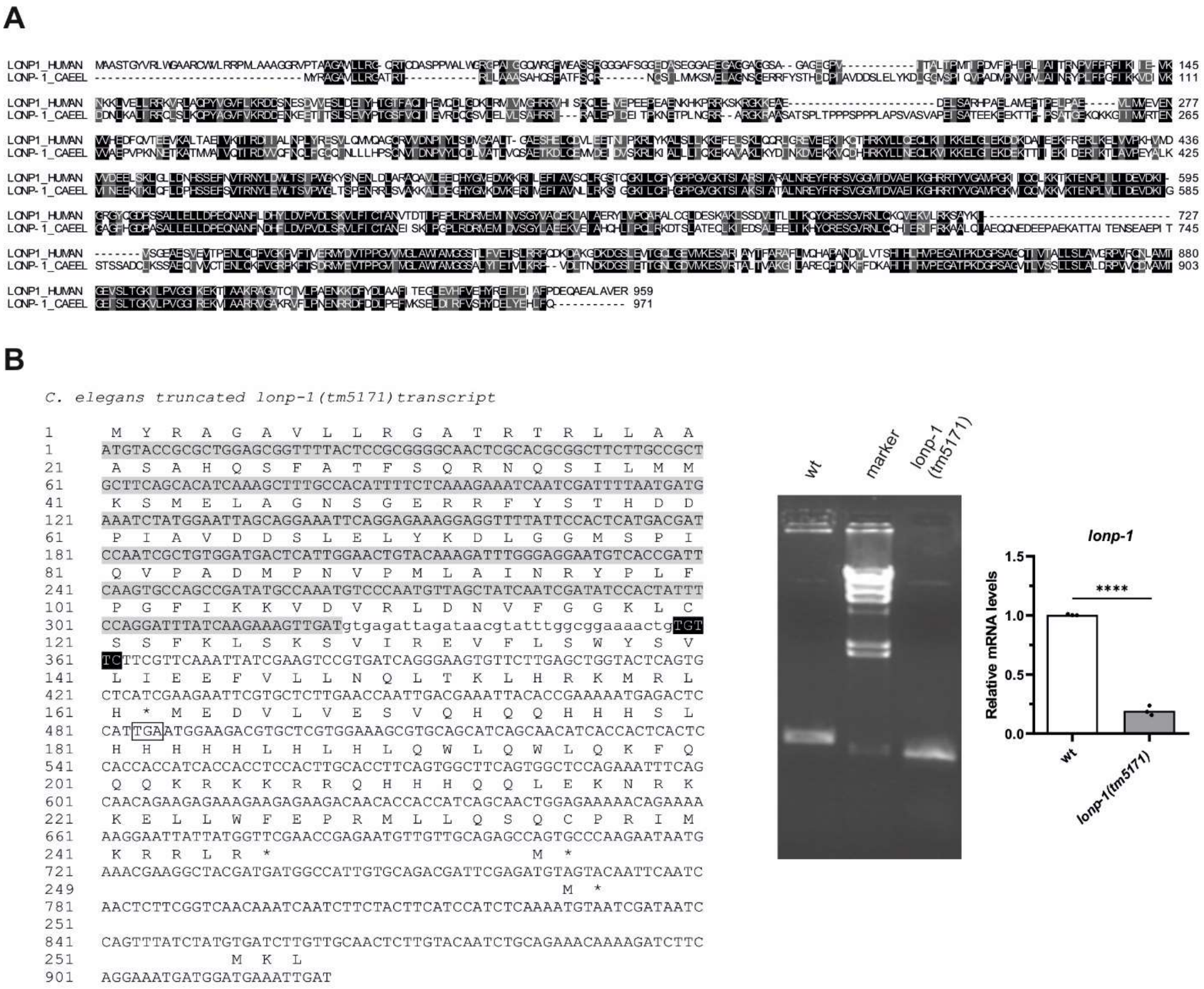

Figure S1. Molecular characterization of *lonp-1(tm5171)* allele. (A) Pair-wise alignment of mitochondrial human (UniProtKB-P36776) and *C. elegans* (UniProtKB-O44952) LonP1 primary amino acid sequences, performed with the EMBOSS Needle Program (https://www.ebi.ac.uk/Tools/psa/emboss_needle/) and Color Align Conservation (http://www.bioinformatics.org/sms2/color_align_cons.html). Black boxes show fully conserved residues. **(B)** The *lonp-1(tm5171)* allele has a 490 bp deletion (shown in Figure 1A) and a 5 bp insertion (black box) which results in a truncated but of low abundance transcript, compared to wt transcript. This was observed in agarose gel and quantitative RT-PCR analyses of the products amplified from cDNA of wt and *lonp-1(tm5171)* worms, and verified by sequence of the related cDNA products. The primers used for PCR and genotyping are reported in Supplementary Table S1. In the expressed mutant transcript, the predicted amino acids that are in common with the wt transcript are marked with grey color (it includes the MTS) and are followed by an altered sequence of 53 amino acids before a premature stop codon (white box).

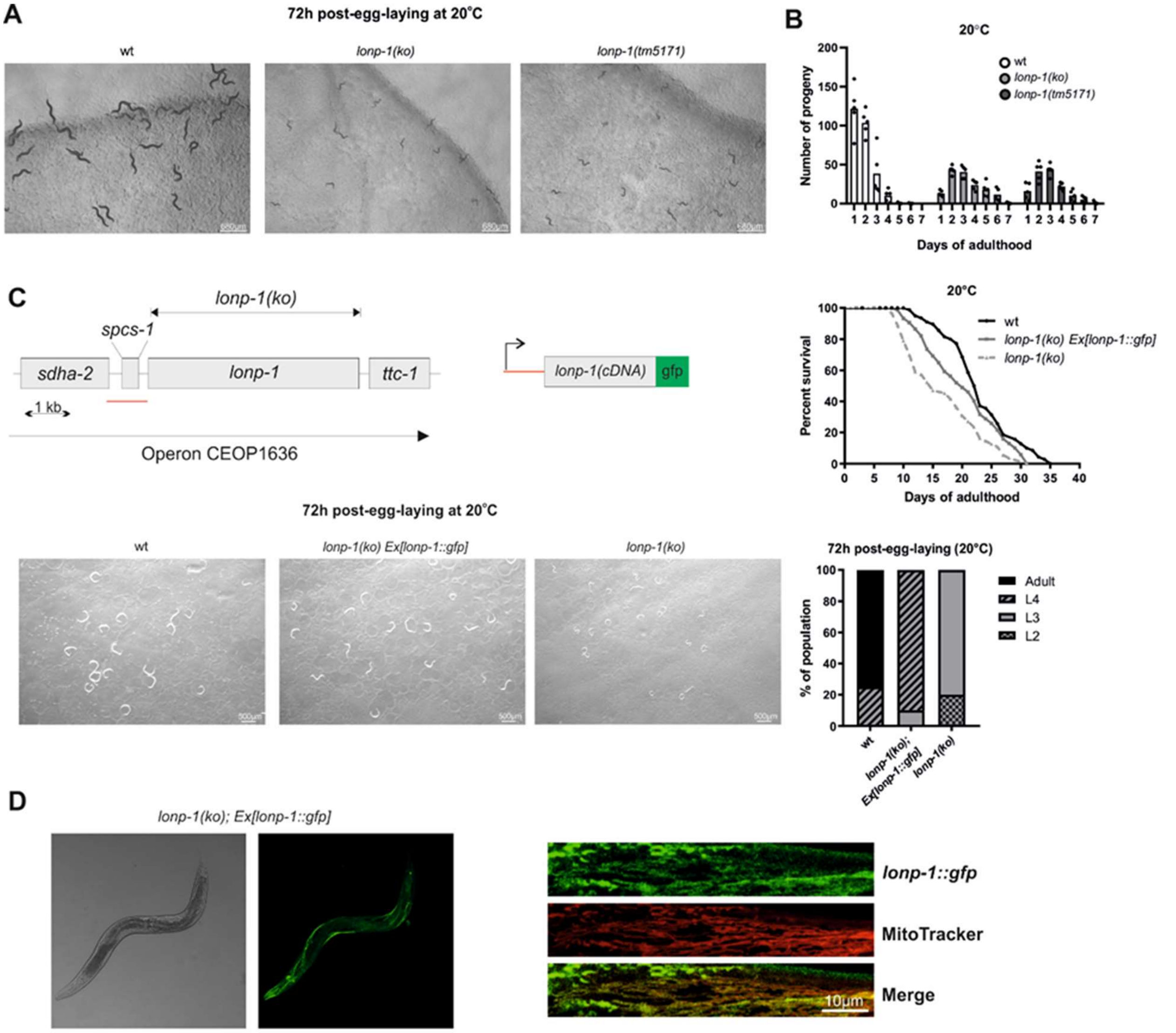

Figure S2. Phenotypic analysis of *lonp-1* mutants and partial rescue of mutant phenotypes by *lonp-1::gfp* transgenes. (A) Photomicrographs of *lonp-1* mutant worms compared to wt, 72 h post-egg-laying at 20°C. Animals of indicated genotypes were observed by stereomicroscopy at the same magnification. The percentage of animals in each developmental stage is shown in Fig. 1C. **(B)** The number of progeny of wt and *lonp-1* adults produced over a period of 7 days, at 20°C. **(C)** Schematic of the generation of a translational *lonp-1::gfp* fusion transgene, the expression of which is driven by an internal promoter in front of *lonp-1* at the operon (shown as a red line). The expressed transgene was able to partially rescue the lifespan and growth defects in *lonp-1(ko)* mutant background (indicating possible lack of sufficient expression level by this internal promoter or a toxic effect from the transgene overexpression). **(D)** A representative microscopy image (brightfield and GFP) of *lonp-1* mutants expressing a *lonp-1::gfp* fusion transgene ubiquitously but of low intensity. Co-localization of the GFP signal with MitoTracker Red CMXRos in a body wall muscle cell. The images shown are Z-stacks of three focal planes collected on a confocal microscope. Scale bar, 10 µm.

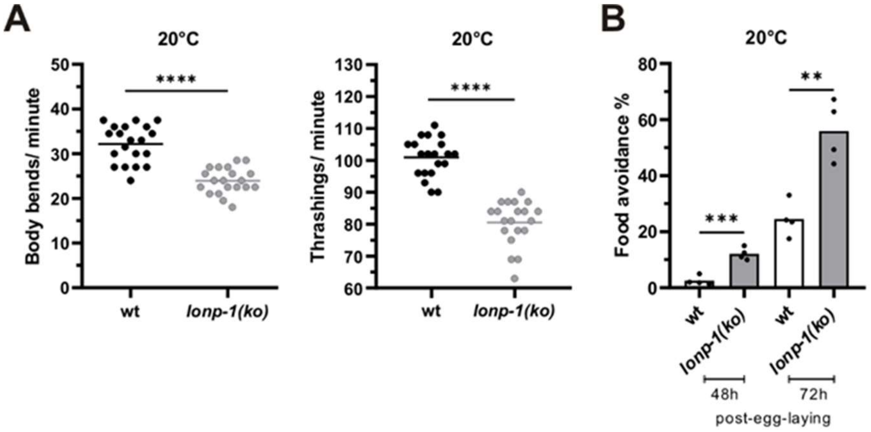

Figure S3. Impaired motility and induction of food-avoidance behavior in *lonp-1* mutants. (A) Motility of 1-day adults as assessed by quantifying the complete body bends on agar plates and the thrashing rate into a liquid medium for 1 min. **(B)** Bacterial-avoidance in wt and *lonp-1* mutant worms, at the indicated time periods. The percentage of "runaway" worms from the lawn is plotted for each plate (N_{off}/N_{total}x100).

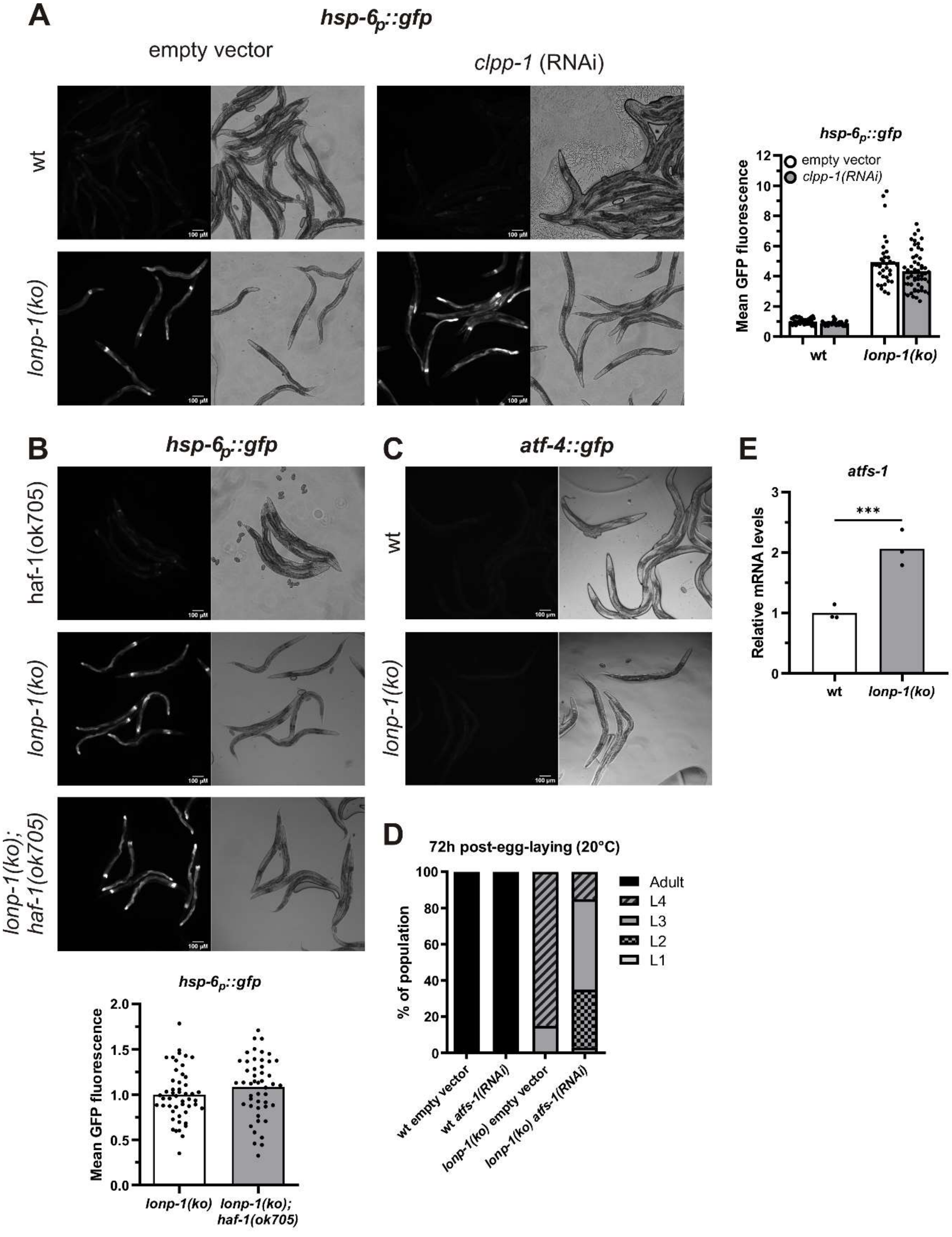

Figure S4. Activation of the UPR^{mt} reporter *hsp-6p::gfp* in *lonp-1* mutant backgrounds are independent of CLPP-1 and HAF-1 factors. (A) Representative microscopy images and GFP fluorescence quantification of *hsp-6p::gfp* transgene, in 1-day adult wt and *lonp-1* worms subjected to *clpp-1(RNAi)* from eggs. **(B)** Representative microscopy images and GFP quantification of *hsp-6p::gfp* transgene, in 1-

day adult wt and *lonp-1* worms carrying a deletion in *haf-1*. Scale bar, 100 μm. **(C)** Representative microscopy images and GFP fluorescence quantification of *atf-4::gfp* transgene, in 1-day adult wt and *lonp-1* worms. **(D)** Growth rate of wt and *lonp-1(ko)* mutant worms fed with *atfs-1(RNAi)*, 72 h post-egg-laying at 20°C, versus the control fed animals. **(E)** Quantification of the relative mRNA levels of endogenous *atfs-1* gene revealed increased expression in *lonp-1* 1-day adults. The normalized mean fold-change of three biological replicates relative to control strain is shown, and significance was assessed by paired *t*-test.

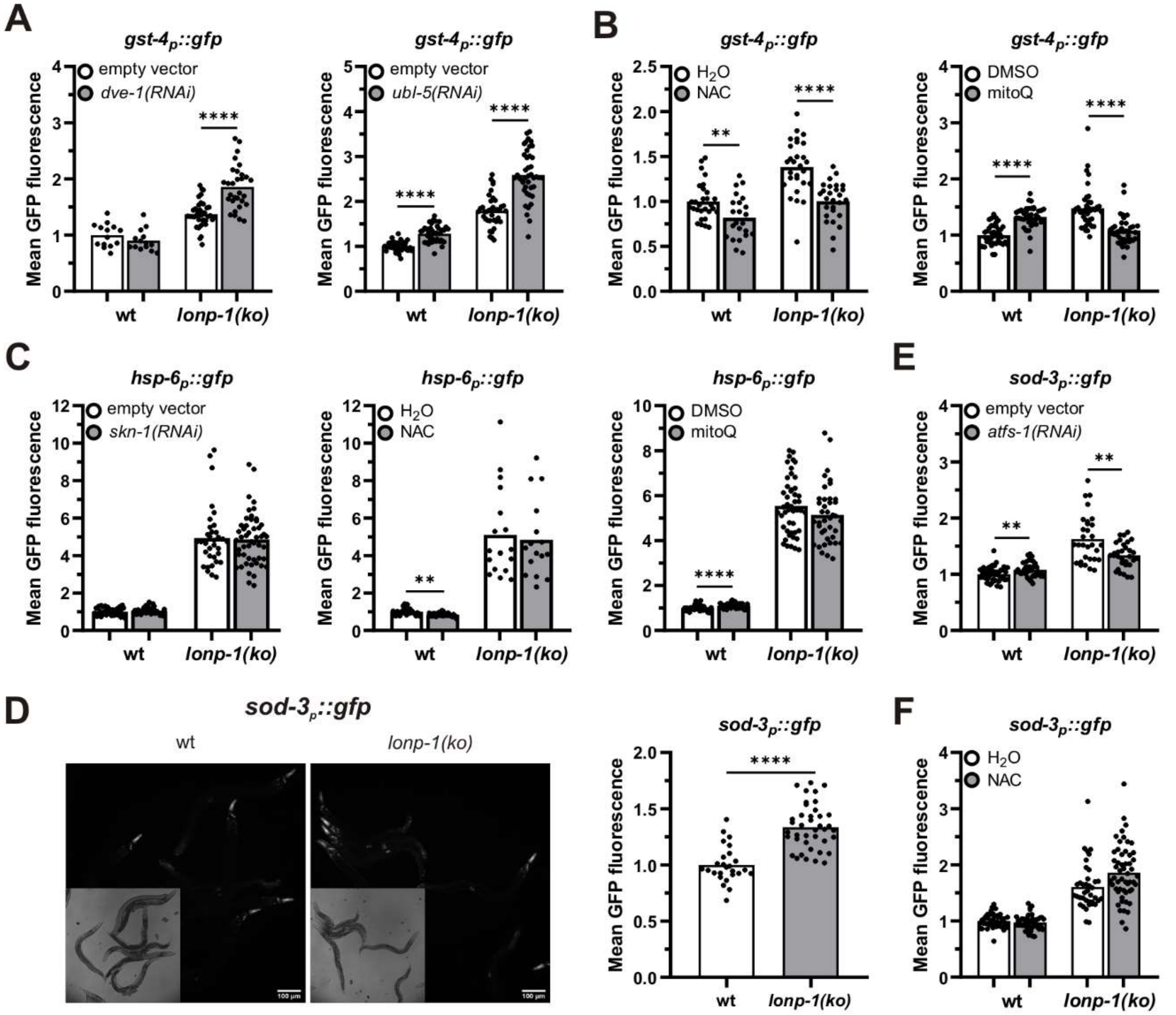

Figure S5. Effect of antioxidant and RNAi treatments on the activation of stress response reporters in *lonp-1* mutants. In all graphs, 1-day adults of wt and *lonp-1(ko)* mutants were used, treated with the indicated RNAi (from eggs) or antioxidant (from the L4 stage for 24 h). The normalized mean GFP fluorescence of worms of all biological replicates was plotted and two-way ANOVA followed by post hoc Tukey's test was used to assess significance of treatment in each strain
(showed as asterisks in each graphs) and the interaction between genotype and treatment (see below). **(A)** Quantification of *gst-*4_p*::gfp* reporter in worms treated with RNAi against *dve-1* or *ubl-5* shows enhanced signal particularly in *lonp-1* mutants and significant interaction (p<0.0001) between genotype and each RNAi. **(B)** Quantification of *gst-*4_p*::gfp* reporter in worms treated with the antioxidant NAC or MitoQ shows significant suppression of fluorescence in *lonp-1* mutants and significant interaction (p=0.0376 for NAC and p<0.0001 for MitoQ). **(C)** Quantification of *hsp-6p::gfp* reporter in worms treated with RNAi against *skn-1* or the antioxidants NAC and MitoQ shows no significant effect in each mutant and no interaction (p=0.6991 for *skn-1(RNAi)*, p=0.8595 for NAC and p=0.0619 for MitoQ treatment). **(D)** Representative microscopy images and GFP quantification of*sod-3p::gfp* transgene, in 1-day adult wt and *lonp-1* worms. The normalized mean fluorescence of all calculated values in three biological

replicates is shown and unpaired *t*-test was used to assess significance. Scale bar, 100 µm. **(E)** Quantification of *sod-3p::gfp* fluorescent reporter in worms treated with RNAi against *atfs-1* shows suppression of signal only in *lonp-1(ko)* and significant interaction (p<0.0001) between genotype and treatment. **(F)** Quantification of *sod-3p::gfp* fluorescent reporter in worms treated with the antioxidant NAC shows increased signal in *lonp-1* mutants and significant interaction (p=0.0078) between genotype and treatment.

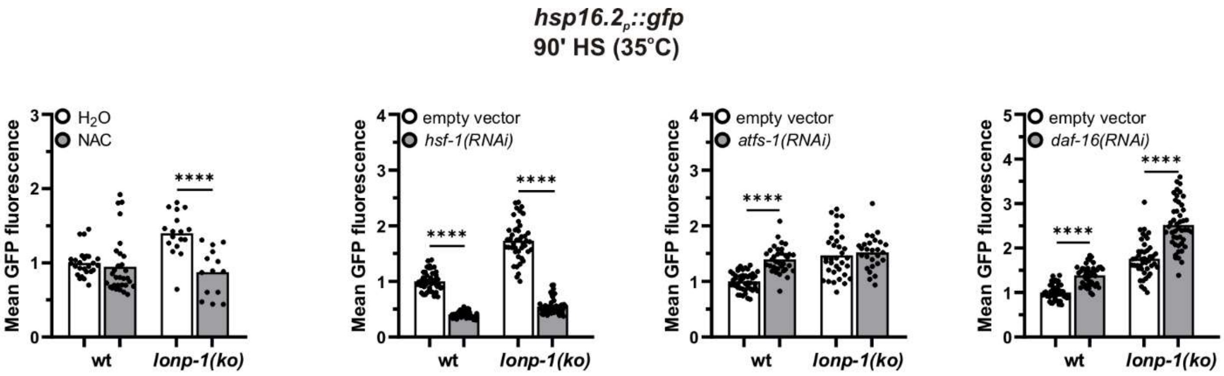

Figure S6. Effect of antioxidant and RNAi treatments on the activation of *hsp-16.2p::gfp* reporter under HS in *lonp-1* mutants. In all graphs, 1-day adults of wt and *lonp-1(ko)* mutants were used, treated with NAC (from the L4 stage for 24 h) or the indicated RNAi (from eggs). The normalized mean GFP fluorescence of worms of all biological replicates was plotted and two-way ANOVA followed by post hoc Tukey's test was used to assess significance of treatment in each strain (showed as asterisks in each graphs) and the interaction between genotype and treatment. Quantification of *hsp-16.2::gfp* reporter in worms treated with the antioxidant NAC shows significant suppression of fluorescence only in *lonp-1* mutants and significant interaction (p=0.001). In both wt and *lonp-1* animals, RNAi against *hsf-1* abolished the induction of *hsp-6p::gfp* reporter, whereas RNAi against *daf-16* further induced the fluorescence. However, in both cases there is significant interaction (p<0.0001 for *hsf-1(RNAi)* and p=0.0007 for *daf-16(RNAi)* between genotype and RNAi. In contrast, RNAi against *atfs-1* has an opposite effect in the induction of the reporter in wt and *lonp-1* worms, with significant interaction (p=0.0016).

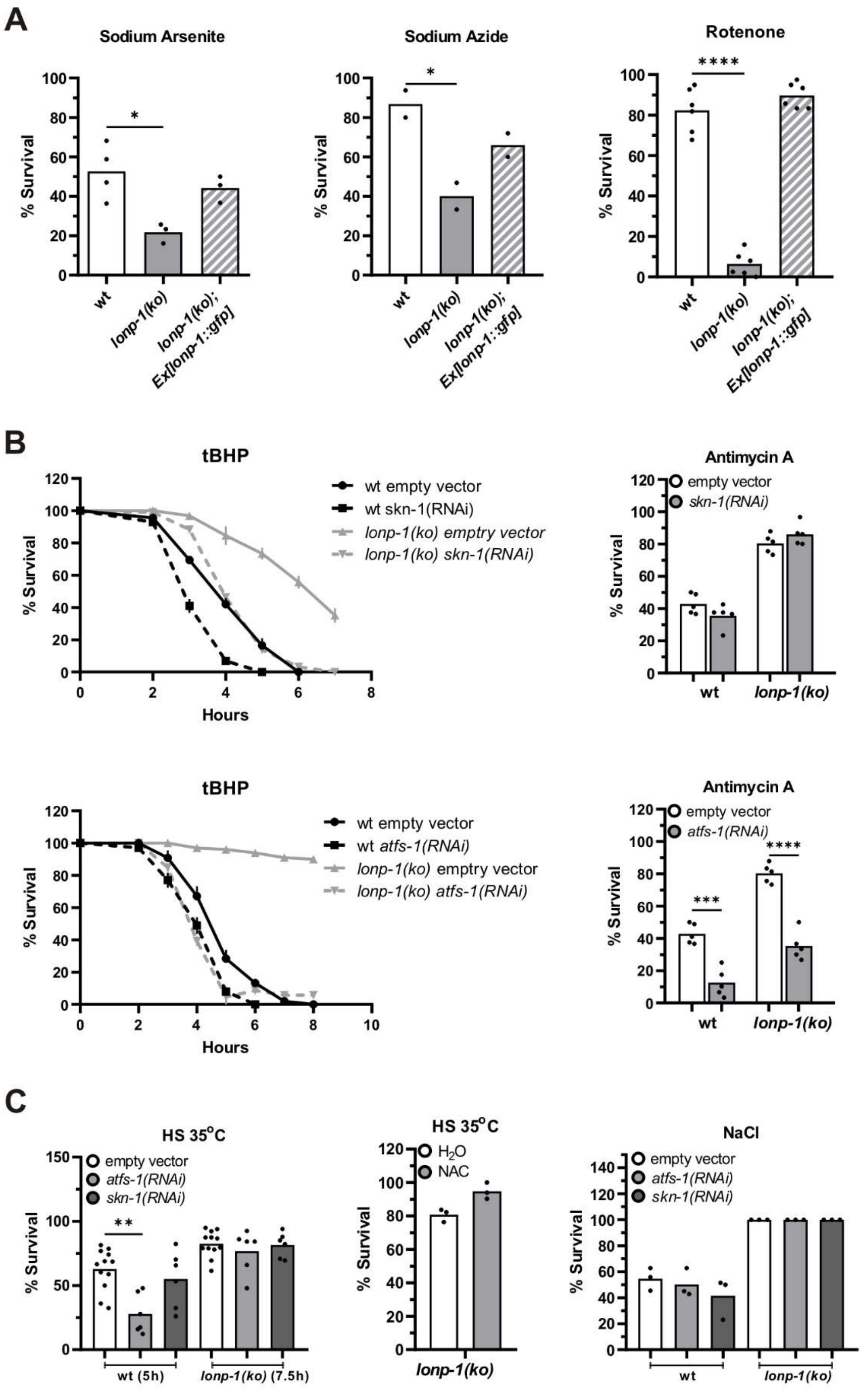

Figure S7. LONP-1 loss induces condition-specific defenses during the organismal oxidative stress response. (A) Expression of LONP-1::GFP transgene rescues the sensitivity of *lonp-1* to

oxidants sodium arsenite (7.5 mM), sodium azide (1.5 mM) and rotenone (10 μm) after 24 h exposure, at day 1 of adulthood. The percentage survival for all biological replicates was plotted and unpaired *t*-test was used to assess significance (*p* value). **(B)** RNAi against *skn-1* significantly impaired survival to tBHP (10 mM) at both wt and *lonp-1(ko)* 1-day adults, whereas *atfs-1(RNAi)* had a dramatic effect only in *lonp-1(ko)* mutants. Two-way ANOVA followed by post hoc Tukey's test was used to assess the interaction between genotype and RNAi treatment, at the time point of 4 h (*p*=0.6849 for *skn-1(RNAi)* and *p*=0.0017 for *atfs-1(RNAi)*). RNAi against *skn-1* had no effect on survival of wt or *lonp-1(ko)* to antimycin A whereas atfs-1(RNAi) significantly reduced survival of both strains. The percentage survival for all biological replicates was plotted and unpaired *t*-test was used to assess significance. **(C)** RNAi against *skn-1* had no effect on survival of wt or *lonp-1(ko)* to heat whereas *atfs-1(RNAi)* made wt animals more sensitive to heat but did not reduce thermotolerance of *lonp-1* mutants. Both RNAi treatments did not affect hypertonic stress resistance and NAC treatment did not compromise thermotolerance of *lonp-1* mutants. The percentage survival for all biological replicates was plotted and unpaired *t*-test was used to assess significance.

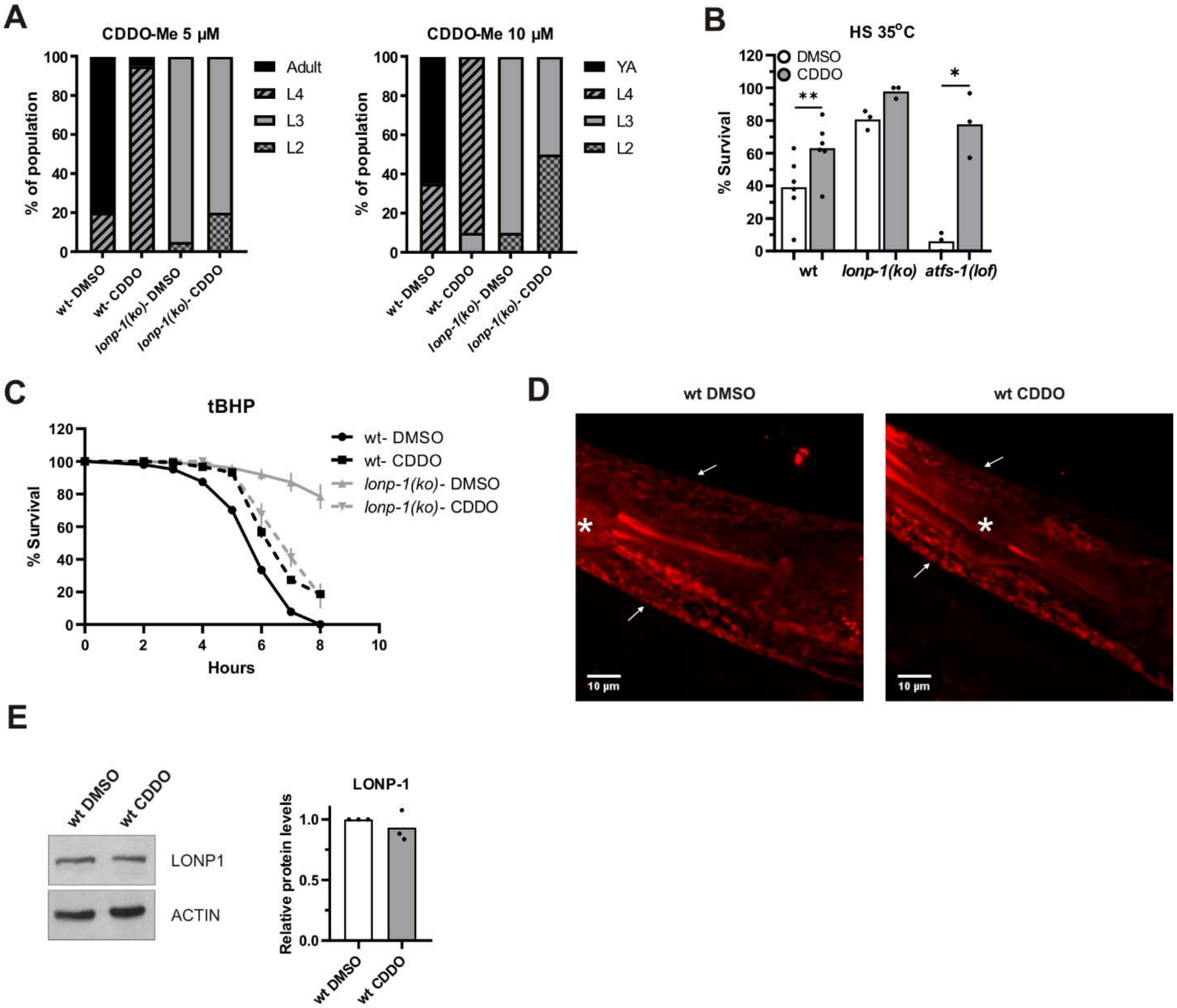

Figure S8. Treatment of wt worms with CDDO-Me impedes growth and enhances stress resistance without affecting mitochondrial network or LONP-1 protein levels. (A) A dose-dependent retardation in growth of both wt and *lonp-1(ko)* mutants treated with CDDO-Me (5 or 10 µM final concentration) compared to the respective solvent concentration (0.05 or 0.1% DMSO final). Eggs were hatched on plates with the indicated chemicals and the percentage of worms in each developmental stage at 72 h post-egg-laying at 20°C is presented. **(B)** Treatment of wt or loss-of-function *atfs-1(gk3094)* mutant worms with 10 µM CDDO-Me from eggs, significantly enhanced their survival to HS (35°C for 5 h), but there was no significant improvement of *lonp-1(ko)* thermotolerance. The percentage survival for all biological replicates was plotted and unpaired *t*-test was used to assess significance. **(C)** Similar treatment with 10 µM CDDO-Me increased survival of wt, but dramatically reduced survival of *lonp-1(ko)* mutants, to oxidative stress induced by tBHP. **(D)** Representative confocal images of vitally stained mitochondria using fluorescent dye Mitotracker Red CMXRos in wt animals treated with 10 µM CDDO-Me or DMSO from eggs. White arrows show intact mitochondrial network in both cases. Asterisk indicates the posterior bulb of the pharynx. Scale bar, 10 µm. **(E)** Western blot analysis and quantification, shows no alterations in LONP-1 protein levels of wt 1-day adults, treated with 10 µM CDDO-Me from eggs. An antibody against human LONP1 and β-Actin as loading control were used.

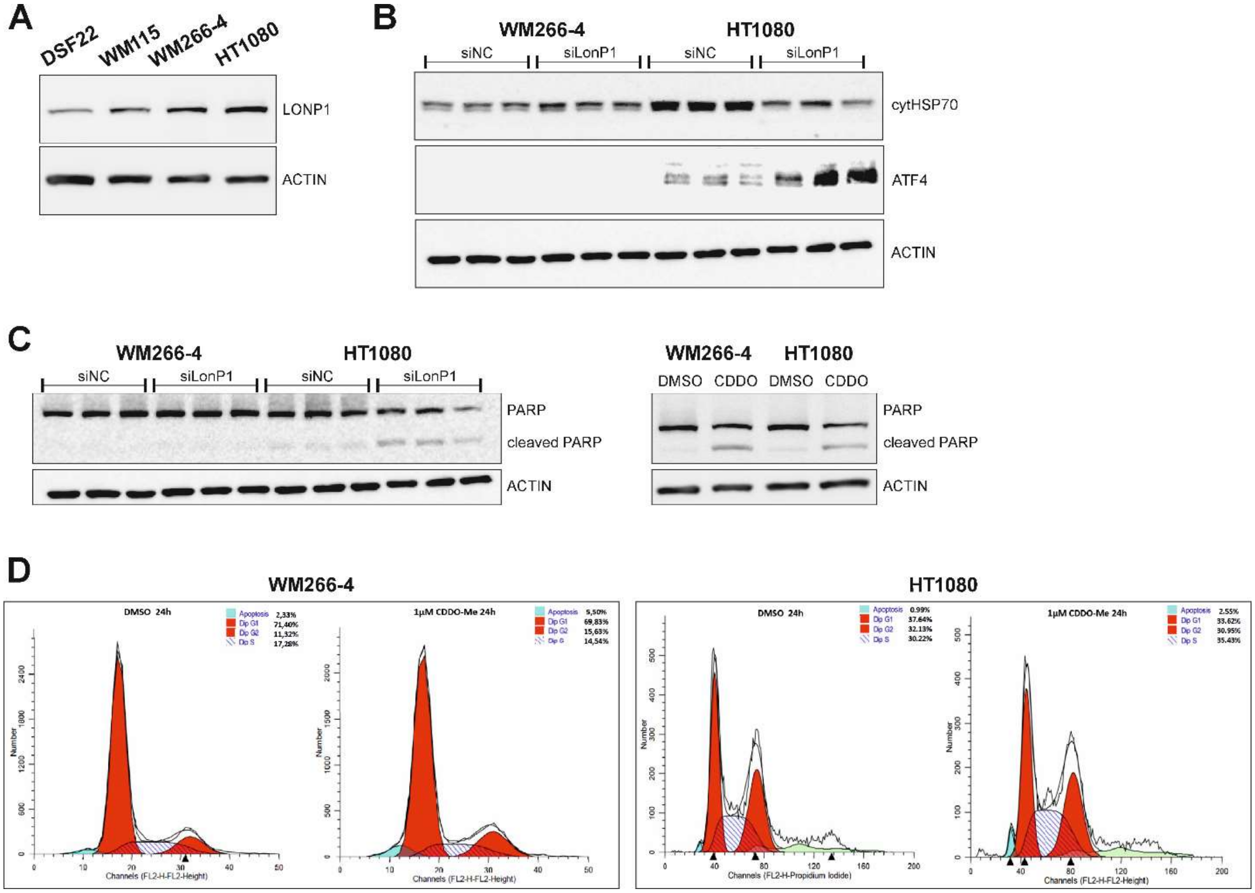

Figure S9. LonP1 protein levels are elevated in cancer cell lines and LonP1 inhibition increases ATF4 protein levels in a cell line-specific manner, causing only mild apoptotic cell death. (A) Western blot analysis of LonP1 and β-Actin using 30μg total protein extracts from normal skin fibroblast (DSF22), primary melanoma (WM115), metastatic melanoma (WM266-4) and fibrosarcoma (HT1080) cell lines. Experiments were repeated three times, while here one representative blot is shown. **(B)** Western blot analysis of HSP70 and ATF4 versus β-Actin upon LonP1 silencing in WM266-4 and HT1080 cell lines. Three biological replicates are represented in the blot. **(C)** Western blot analysis of PARP (116 kDa) and its cleaved form (89 kDa) versus β-Actin upon LonP1 silencing or 1 μM CDDO-Me treatment for 24 h, in both cell lines. Three biological replicates are represented in the blot. **(D)** Cell cycle progression analysis of WM266-4 and HT1080 cells treated with 1 μM CDDO-Me for 24 h. Representative PI/FACS analysis and percentage of total cell content in distinct cell cycle phases as wells as the content in apoptotic cells are displayed.
