# Supplementary material for: Organismal and Cellular Stress Responses upon Disruption of Mitochondrial Lonp1 Protease
Source: Cells. 2022 Apr 16;11(8):1363. doi: 10.3390/cells11081363 (PMC9025075; doi:10.3390/cells11081363)
Supplement: Supplementary file 1 [file cells-11-01363-s001.zip › cells-1658543-supplementary/Table S1.pdf]

Table S1. List of *C. elegans* strains used in this study.

Strain	Genotype	Information	Reference
N2	N2 (Bristol)	Wild-type (wt)	CGC
BRF366	N2;*synEx31[rol-6(su1006)]*	Injection of pRF4 in wt. Pick rollers	This study
FX14710	*lonp-1(tm5171)I*	Indel allele of *lonp-1* (C34B2.6)	NBRP
BRF548	*lonp-1(tm5171)I (OC)*	Outcrossed 5 times with N2	This study
BRF791	*lonp-1(ko)I (OC)*	Outcrossed 5 times witn N2, CRISPR/Cas9-mediated knockout of *lonp-1*	This study
BRF367	*lonp-1(ko)I*;*synEx31[rol-6(su1006)]*	Cross of BRF791 males with BRF366 hermaphrodites. Pick rollers	This study
BRF611	*lonp-1(ko)I;synEx455[lonp-1_p::lonp-1::gfp;rol-6(su1006)]*	*lonp-1* rescue strain. Injection of *lonp-1p::lonp-1::gfp* (cloned in pPD95.77 vector) in *lonp-1(ko)* mutants. Pick rollers	This study
SJ4103	*zcIs14[myo-3_p::gfp(mit)]*	Stable transgenic line expressing GFP in mitochondria of body wall muscle	CGC
BRF811	*lonp-1(ko)I;zcIs14[myo-3_p::gfp(mit)]*	Cross of BRF791 males with SJ4103 hermaphrodites	This study
JV1	*unc-119(ed3)III;jrls1[rpl-17p::HyPer+unc119(+)]*	Stable transgene ubiquitously expressing the H_2O_2 sensor HyPer	CGC
BRF802	*lonp-1(ko)I;jrls1[rpl-17p::HyPer+unc119(+)]*	Cross of BRF791 males with JV1 hermaphrodites	This study
SJ4100	*zcIs13[hsp-6_p::gfp, lin-15(+)]V*	Stable transgenic line with GFP expression mainly in the tail. Used as UPR^{mt} marker	CGC

Strain	Genotype	Information	Reference
BRF789	*lonp-1(ko)I*; zcIs13(*hsp-6$_p$::gfp, lin-15(+)]V*	Cross of BRF791 males with SJ4100 hermaphrodites	This study
BRF786	*lonp-1(tm5171)I*; zcIs13(*hsp-6$_p$::gfp, lin-15(+)]V*	Cross of BRF548 males with SJ4100 hermaphrodites	This study
SJ4058	*zcIs9[hsp-60$_p$::gfp, lin-15(+)]V*	Stable transgenic line with low GFP expression, mainly in the tail. Used as UPR^{mt} marker	CGC
BRF790	*lonp-1(ko)I;zcIs9 hsp-60$_p$::gfp, lin-15(+)]V*	Cross of BRF791 males with SJ4058 hermaphrodites	This study
BRF787	*lonp-1(tm5171);hsp-60$_p$::gfp, lin-15(+)]V*	Cross of BRF548 males with SJ4058 hermaphrodites	This study
CF1407	*daf-16(mu86)I;muIs71[daf-16a$_p$::GFP::daf-16a); rol-6(su1006)]X*	Stable line expressing the GFP::DAF-16a protein in both cytoplasm and nuclei of neurons, muscles, intestinal cells and hypodermis	CGC
BRF801	*lonp-1(ko)I;muIs71[daf-16a$_p$::gfp::daf-16a);rol-6(su1006)]X*	Cross of BRF791 males with CF1407 hermaphrodites	This study
CL2070	*dvIs70[hsp-16.2$_p$::gfp;rol-6(su1006)]*	Stable line with robust induction of *gfp* after heat-shock. Used as HSR marker	CGC
BRF809	*lonp-1(ko)I;dvIs70[hsp-16.2$_p$::gfp;rol-6(su1006)]*	Cross of BRF791 males with CL2070 hermaphrodites	This study
RB867	*haf-1 (ok705)IV*	Deletion of *haf-1*	CGC

Strain	Genotype	Information	Reference
BRF820	*lonp-1(ko)I;haf-1(ok705)IV*	Cross of BRF791 males with RB867 hermaphrodites	This study
BRF767	*haf-1(ok705)IV;zcIs13[hsp-6$_p$::gfp, lin-15(+)]V*	Cross of BRF820 males with SJ4100 hermaphrodites	This study
BRF816	*lonp-1(ko)I;haf-1(ok705)IV; zcIs13[hsp-6$_p$::gfp, lin-15(+)]V*	Cross of BRF820 males with SJ4100 hermaphrodites	This study
CL2166	*dvIs19[(gst-4$_p$::gfp::NLS] III*	Stable line used as oxidative stress marker	CGC
BRF813	*lonp-1(ko)I;dvIs19[gst-4$_p$::gfp::NLS]III*	Cross of BRF791 males with BRF813 hermaphrodites	This study
CF1553	*muIs84[sod-3$_p$::gfp;rol-6(su1006)]*	Stable line with gfp expression in head, tail and around vulva.	CGC
BRF105	*lonp-1(ko)I;muIs84[sod-3$_p$::gfp;rol-6(su1006)]*	Cross of BRF791 males with BRF105 hermaphrodites	This study
VC3201	*atfs-1(gk3094)V*	Loss-of-function allele of ZC376.7	CGC
GR2250	*mgIs73 [cyp-14A4p::gfp::cyp-14A 3'UTR+myo-2p::mCherry]*	Stable line with gfp expression predominantly in the intestine. Used as marker of mitochondrial dysfunction.	CGC
BRF139	*lonp-1(KO); mgIs73 [cyp-14A4p::gfp::cyp-14A 3'UTR+myo-2p::mCherry]*	Cross of BRF791 males with GR2250 hermaphrodites	This study
BRF140	*N2;Ex[ATF-4::GFP;pRF4 rol-6(su1006)]*	Translational fusion of T04C10.4 under its promoter. Used as ISR marker	[63]

**Strain**	**Genotype**	**Information**	**Reference**
BRF855	*lonp-1(ko)I;Ex[ATF-4::GFP;pRF4 rol-6(su1006)]*	Cross of BRF791 males with BRF140 hermaphrodites	This study
