# Supplementary material for: Organismal and Cellular Stress Responses upon Disruption of Mitochondrial Lonp1 Protease
Source: Cells. 2022 Apr 16;11(8):1363. doi: 10.3390/cells11081363 (PMC9025075; doi:10.3390/cells11081363)
Supplement: Supplementary file 1 [file cells-11-01363-s001.zip › cells-1658543-supplementary/Table S2.pdf]

Table S2. List of primers used in this study.

Primer name	Oligonucleotide sequences (5'→3')	Target
Primers for *C. elegans*		
CD.Cas9.LON.Frw	ACGTTTTACTCGGGATCTGGT	*lonp-1(ko)* by CRISPR/Cas9
CD.Cas9.LON.Rev	ATCGACAAGCTTCAAACCGC	
Lon Frw	CGTTTTACTCGGGATCTGGTC	Tracking *lonp-1 mutants*
Lon Rev	GATATTACCGAATCGTCTGCAC	
Lon ko Frw	TTAACCGGTCCATGTCACCGATTCAAGTGCC	
Lon ko Rev	GCTTCCACAGCAGATTCCC	
haf-1 Frw	GGTGGCGGCAGATGTTAATC	Tracking *haf-1(ok705)*
haf-1 Rev	CCGACAAATTTTCTCGCCCT	
atfs-1 Frw	ATGTTTTCCCGTGTGGGACGT	Tracking *atfs-1(gk3094)*
atfs-1 Rev	CGAACATTTTTCCGTGAAGATAACT	
atfs-1 Frw	ATGTTTTCCCGTGTGGGACGT	*atfs-1*(qRT-PCR)
atfs-1 RT2	TCCGAAGCTGGTCGTTCTATG	
daf-21 RT1	TCTCGAAATCAACCCAGACC	*daf-21* (qRT-PCR)
daf-21 RT2	CTCAGCTCCCTCAATCTTGG	
gst-4 RT1	TGCTCAATGTGCCTTACGAG	*gst-4* (qRT-PCR)
gst-4 RT2	AGTTTTTCCAGCGAGTCCAA	
gst-13 RT1	CAAATTCCCCAATCTATGGCAATTG	*gst-13* (qRT-PCR)
gst-13 RT2	TCCAAGCAAGAATCACTGGATAATC	
hsf-1 RT1	GCATAACAATATGAATAGCATGG	*hsf-1* (qRT-PCR)
hsf-1 RT2	GACGTCCTTGTACAAAACACGG	
hsp-1 RT1	TGACAAGTCCACCGGAAAGC	*hsp-1* (qRT-PCR)

hsp-1 RT2	TGATCTTATCCTTGAGCTTCTCG	
hsp-3 RT1	CATTGCCAACGACCAAGGAAAC	*hsp-3* (qRT-PCR)
hsp-3 RT2	AATGGCCAGTGCTTGATGTCAG	
hsp-4 RT1	TGGCAAACGCGTACTGTGATG	*hsp-4* (qRT-PCR)
hsp-4 RT2	CGCAACGTATGATGGAGTGAT	
hsp-16.1 RT1	CTCCATCTGAATCTTCTGAGATTG	*hsp-16.1* (qRT-PCR)
hsp-16.1 RT2	CTTGAATTGATAATGTATGTCCATCC	
hsp-16.2 RT2	CCTTGAACCGCTTCTTTCTTTG	*hsp-16.2* (qRT-PCR)
hsp-16.2 RT3	CCATCTGAGTCTTCTGAGATTGTT	
hsp-6 RT1	GGTTCAAAAGGACTTAAAGGTCG	*hsp-6* (qRT-PCR)
hsp-6 RT2	GTTGTTGACGGTGGTTCCC	
hsp-60 RT1	AGATCGAGAAGCGAATCGAG	*hsp-60* (qRT-PCR)
hsp-60 RT2	GGTGACACGGTCCTTCTTCT	
hsp-70 RT1	TTCAATGGGAAGGACCTCAA	*hsp-70* (qRT-PCR)
hsp-70 RT2	CCTCCAGCTGTTTCAATTCC	
F44E5.4 RT1	CAGAATGGAAAGGTTGAGATCCTC	*hsp-70* (qRT-PCR)
F44E5.4 RT2	CATCGAAACGTCGTCCAATCAATC	
lonp-1 RT3	CATGTACCCGAAGGAGCAA	*lonp-1* (qRT-PCR)
lonp-1 RT4	GCGATCACTTTTTCCCGAAT	
mtl-1 RT1	ATGGCTTGCAAGTGTGACTG	*mtl-1* (qRT-PCR)
mtl-1 RT2	GCTTCTGCTCTGCACAATGA	
skn-1 RT1	AAAGTTGGCTCCATCCAGTG	*skn-1* (qRT-PCR)
skn-1 RT2	ACCTTGTTCTTTCCGCGTCG	
sod-3 RT1	TCGGTTCCCTGGATAACTTG	*sod-3* (qRT-PCR)
sod-3 RT2	CATAGTCTGGGCGGACATTT	

atfs-1 Frw	ATGTTTTCCCGTGTGGGACGT	*atfs-1* (RNAi construct)
atfs-1 Rev	CGAACATTTTTCCGTGAAGATAACT	
clpp-1i Frw	TTAAGCTTATGATAAGTGCACCAGTGTCC	*clpp-1* (RNAi construct)
clpp-1i Rev	CATGGATCCTTTAATCTGATGGCATTGATCC	
daf-16i Frw	CAATGAGATTTATCAATGGTTCTC	*daf-16α* (RNAi construct)
daf-16i Rev	CGATTGAGTTCGGGGACTG	
dve-1i Frw	ATCTAGACACTCTTTACGAAATTCCACG	*dve-1* (RNAi construct)
dve-1i Rev	ACTCGAGTGGGTCGAACATCAGG	
hsf-1i Frw	CCACCGGTATGCAGCCAACAGGGAATC	*hsf-1* (RNAi construct)
hsf-1i Rev	GGCTCGAGTTAAACCAAATTAGGATCCGATGG	
lonp-1i Frw	TTAACCGGTAATGTACCGCGCTGGAGC	*lonp-1* (RNAi construct)
lonp-1i Rev	GCGATCACTTTTTCCCGAAT	
skn-1i Frw	GGGAAGCTTCCAACTACGCCTACATTGG	*skn-1* (RNAi construct)
skn-1i Rev	GGGCTCGAGCTTGTCGTGACGATCCGTG	
ubl-5i Frw	TTTGAAGAAGTTGATCGCTGC	*ubl-5* (RNAi construct)
ubl-5i Rev	TGAATCCCTCGTGAATCTCG	
Primers for cell lines		
LonP1 Frw	AAGACCATTGCGGCCAAG	*LONP1* (qRT-PCR)
LonP1 Rev	GGAAGGCGATGTCGAAGAT	
ATF5 Frw	TGCTGCAGCCATGGAGTC	*ATF5* (qRT-PCR)
ATF5 Rev	GGTCGCCAGGAGTGACAT	
ATF4 Frw	CAGCAAGGAGGATGCCTTC	*ATF4* (qRT-PCR)
ATF4 Rev	CAGGTCATCTATACCCAACAG	
CHOP Frw	CTCCTGGAAATGAAGAGGAAGA	*DDIT3* (qRT-PCR)
CHOP Rev	TGTGACCTCTGCTGGTTCTG	

mtHsp70 Frw	CAAGCGACAGGCTGTCACCAAC	*HSPA9* (qRT-PCR)
mtHsp70 Rev	CAACCCAGGCATCACCATTGG	
Hsp60 Frw	ATTGACCCAACAAAGGTTGTGAG	*HSPD1* (qRT-PCR)
Hsp60 Rev	CATACCACCTCCCATTCCAC	
TRAP-1 Frw	CAGCTGCTGGTGGATCAGATA	*TRAP1* (qRT-PCR)
TRAP-1 Rev	TGTGGTGTCAGTCCTTCTGG	
NRF2 Frw	TCCATTCCTGAGTTACAGTGTCTT	*NFE2L2* (qRT-PCR)
NRF2 Rev	TCTTTTTCCATTGAGGGTATAGATG	
HO-1 Frw	CTGCTCAACATCCAGCTCTTT	*HMOX1* (qRT-PCR)
HO-1 Rev	GGGCAGAATCTTGCACTTTG	
Hsp90α Frw	TTAGTGTCAGTCACCAAAGAAGG	*HSP90AA1* (qRT-PCR)
Hsp90α Rev	ACAATACAGCATGGAGATGTCAC	
Hsp70 Frw	CCAGATCGAGGTGACCTTC	*HSPA1A* (qRT-PCR)
Hsp70 Rev	TTGTACTTCTCCGCCTCCTG	
Hsc70 Frw	GAAGATTCTGGACAAGTGTAATG	*HSPA8* (qRT-PCR)
Hsc70 Rev	TGGTACAGCTTGGTGATGATG	
CRYAB Frw	CCCAGAGGAACTCAAAGTTAAG	*CRYAB* (qRT-PCR)
CRYAB Rev	CCATTCACAGTGAGGACCC	
Hsp27 Frw	GTCCCTGGATGTCAACCACT	*HSPB1* (qRT-PCR)
Hsp27 Rev	GATGTAGCCATGCTCGTCCT	
GADD34 Frw	GCCCAGAAACCCCTACTCA	*PPP1R15A* (qRT-PCR)
GADD34 Rev	TGCCCAGACAGCCAGGAAA	
ACTB Frw	GATCAAGATCATTGCTCCTCCTG	*ACTB* (qRT-PCR)
ACTB Rev	CGTCATACTCCTGCTTGCTG	
