# Supplementary material for: Organismal and Cellular Stress Responses upon Disruption of Mitochondrial Lonp1 Protease
Source: Cells. 2022 Apr 16;11(8):1363. doi: 10.3390/cells11081363 (PMC9025075; doi:10.3390/cells11081363)
Supplement: Supplementary file 1 [file cells-11-01363-s001.zip › cells-1658543-supplementary/Table S3.pdf]

Table S3. Analysis of lifespan assay results.

Strain	Genotype	Median/ Max	Temperature (ºC)	Mean/SEM	n(T/C)	p-value vs control strain	p-value vs empty vector
N2	Wild-type (wt)	24/29	20[a,b,c]	23.33/ 0.3333	135/6	-	-
BRF336	*lonp-1(tm5171)*	19/27		19.33/ 0.3333	126/25	<0.0001(****)	-
BRF791	*lonp-1(ko)*	20/29		20.00/ 0.5774	106/12	<0.0001(****)	-
BRF638	wt;rol-6(*su1006)*	23/34	20[a,d]	22.67/ 0.8819	92/21	-	-
BRF611	*lonp-1(ko); rol-6(su1006)*	15/29		14.67/ 1.453	107/45	<0.0001(****)	-
BRF663	*lonp-1(ko); lonp-1::gfp; rol-6(su1006)*	20/31		19.67/ 1.667	94/24	0.0201(*) vs wt 0.0059(**) vs *lonp-1(ko)*	-
N2	wt	21/32	20[a]	21.33/ 0.1667	92/24	-	-
BRF336	*lonp-1(tm5171)*	16/30		16.33/ 1.667	95/41	<0.0001(****)	-
N2	wt	19/26	20[a]	19.67/ 0.3333	110/4	-	-
BRF791	*lonp-1(ko)*	14/24		14.00/ 0.5774	113/5	<0.0001(****)	-

Strain	Genotype	Median/ Max	Temperature (ºC)	Mean/SEM	n(T/C)	p-value vs control strain	p-value vs empty vector
N2	wt	20/27	20[a]	19.67/ 0.3333	133/19	-	-
BRF791	*lonp-1(ko)*	15/26		15.00/ 0.5774	125/18	<0.0001(****)	-
N2	wt	11/21	25[a]	10.67/ 0.3333	131/2	-	-
BRF791	*lonp-1(ko)*	8/22		8.167/ 0.1667	100/1	<0.0001(****)	-
N2	wt	14/19	25[a]	13.33/ 0.3333	125/13	-	-
BRF791	*lonp-1(ko)*	9/19		9.000/ 0.000	90/17	<0.0001(****)	-
N2	wt	14/21	25[a]	13.67/ 0.3333	106/12	-	-
BRF791	*lonp-1(ko)*	9/21		8.500/ 0.5000	77/8	0.0124(*)	-
N2 empty vector	wt	18/30	20[a,e,f]	18.00/ 0.9129	147/8	-	-
N2 *atfs-1(RNAi)*		19/31		19.25/ 0.7500	152/5	-	0.0788(ns)

Strain	Genotype	Median/ Max	Temperature (ºC)	Mean/SEM	n(T/C)	p-value vs control strain	p-value vs empty vector
BRF791 empty vector	*lonp-1(ko)*	19/30		19.25/ 0.2500	126/8	0.8616(ns)	-
BRF791 *atfs-1(RNAi)*		16/28		16.33/ 0.3333	107/9	0.0001(***)	0.0160(*)
N2 empty vector	wt	17/27	20[a,e,f]	17.33/ 0.3333	111/1	-	-
N2 *atfs-1(RNAi)*		17/27		16.67/ 0.6667	129/0	-	0.7433(ns)
BRF791 empty vector	*lonp-1(ko)*	19/29		19.00/ 0.5774	111/1	0.0559(ns)	-
BRF791 *atfs-1(RNAi)*		18/28		17.67/ 0.3333	105/0	0.9992(ns)	0.0276(*)
N2 empty vector	wt	20/28	20[a,e,f]	20.00/ 0.000	116/5	-	-
N2 *atfs-1(RNAi)*		20/28		20.17/ 0.4410	124/1	-	0.5031(ns)

Strain	Genotype	Median/ Max	Temperature (ºC)	Mean/SEM	n(T/C)	p-value vs control strain	p-value vs empty vector
BRF791 empty vector	*lonp-1(ko)*	21/28		21.33/ 1.202	125/11	0.1050(ns)	-
BRF791 *atfs-1(RNAi)*		20/27		20.00/ 0.5774	123/3	0.3682(ns)	0.0202(*)

[a] In OP50 plates

[b] In FUDR plates (10 mg/ml) with dead bacteria from day-1 of adulthood

[c] Figure 1E in the text

[d] Supplementary figure 2C in the text

[e] In HT115(DE3) bacteria

[f] Post-developmental RNAi (picked as L4s from OP50)
